# Super-gain nanostructure with self-assembled well-wire complex energy-band engineering for high performance of tunable laser diodes

**DOI:** 10.1515/nanoph-2023-0013

**Published:** 2023-03-27

**Authors:** Yuhong Wang, Hanxu Tai, Ruonan Duan, Ming Zheng, Wei Lu, Yue Shi, Jianwei Zhang, Xing Zhang, Yongqiang Ning, Jian Wu

**Affiliations:** School of Physics, Beihang University, Beijing 102206, China; State Key Laboratory of Luminescence and Application, Changchun Institute of Optics, Fine Mechanics and Physics, Chinese Academy of Sciences, Changchun 130033, China

**Keywords:** indium-segregation effect, multi-atomic step effect, optical gain, semiconductor nanostructure, tunable laser diodes

## Abstract

Although traditional quantum-confined nanostructures e.g. regular quantum wells or quantum dots have achieved huge success in the field of semiconductor lasers for past decades, these traditional nanostructures are encountering the difficulty of enhancing device performance to a higher level due to their inherent gain bottleneck. In this paper, we are proposing a new super-gain nanostructure based on self-assembled well-wire complex energy-band engineering with InGaAs-based materials to break through the existing bottleneck. The nanostructure is constructed by utilizing the special strain-driven indium (In)-segregation and the growth orientation-dependent on-GaAs multi-atomic step effects to achieve the distinguished ultra-wide and uniform super-gain spectra. The structural details and its luminescence mechanism are investigated by multiple measurement means and theoretical modeling. The polarized gain spectra with the max fluctuation of <3 cm^−1^ in 904 nm–998 nm for transverse electric (TE) mode and 904 nm–977 nm for transverse magnetic (TM) mode are simultaneously obtained with this nanostructure. It enables an ultra-low output power fluctuation of <0.7 dB and a nearly-constant threshold power throughout an ultra-wide wavelength range under a fixed injection level. It was difficult to realize these in the past. Therefore, the described super-gain nanostructure brings a brand-new chance of developing high performance of tunable laser diodes.

## 1 Introduction

The traditional quantum-confined nanostructures that are usually used in laser diodes, e.g. regular quantum wells, wires or dots of III–V compounds and their combinations have played an important role in the development of semiconductor lasers for the past decades [1–7]. However, with the rapid development of industrial applications, these relatively-simple nanostructures have no longer been able to meet increasing requirements of the industrial application due to their inherent bottlenecks. For example, these traditional quantum-confined nanostructures can only generate very limited bandwidth and non-uniform distribution of optical gains, when they are used as active materials of the laser diodes (see Figure 7e) [8–10]. This has become a fatal shortcoming for tunable laser diodes, as a very wide and uniform gain spectrum is always expected for the tunable lasers to obtain an ultra-wide wavelength-tuning range with the identical output power under a fixed injection power level. Unfortunately, such a high performance of tunable laser diodes has not been realized so far (see Figure 8c and d).

In order to obtain the tunable laser diodes with the high performance mentioned above, some approaches have been investigated. One type of them uses a more complex cavity, in which more than one gain chips with staggered gain peaks are combined to obtain a broader wavelength-tuning range and more identical output power based on the gain superposition effect [11–13]. However, the effect is very limited with this approach. The other type of the approaches is the combination or coupling of several quantum-confined structures with different band-gaps in one chip to reach the goal of increasing the gain bandwidth based on the gain superposition, as well [14–18]. However, it is still unsatisfying to obtain both wide and uniform super-gain spectra, as a finite quantity of quantum-confined structures can be used in one chip.

The uses of multiple technical means and new materials in design and processing of micro/nanostructures have brought some unique advantages in the improvement of applied material properties [19–21]. These provide a new idea for us to enhance the performance of tunable laser diodes by optimizing the medium structure. Recently, some complex nanostructures based on the combination of quantum wells and dots have attracted much attention and investigation [22–25]. In such a complex nanostructure, the well can be used as a container of gathering and storing carriers for producing high output power and the dots are used as the accessory of transporting the carriers and expanding the gain spectrum to the long-wavelength direction for the tunable laser diodes. However, the shortcomings of the quantum dots are also clear. On the one hand, the dots are poor in the energy storage and it is also difficult to obtain regular dots in both size and distribution from the material growth [26–28]. On the other hand, the very sharp and narrow gain profile of the dots also makes it difficult to achieve a very uniform super-gain spectrum by gain superposition of the well and the dots [29].

These previous effort and issues make us to think about a new structural design based on special energy-band engineering to achieve the super-gain nanostructure for tunable laser diodes. It is a sort of self-assembled well-wire complex nanostructure, which can be formed through the strain-driven indium (In)-segregation and the growth orientation-dependent on-GaAs multi-atomic step effects. Theoretically, the wider gain bandwidth of the wires than the dots makes it more possible to obtain both wide and uniform super-gain spectra from the gain superposition of well and wires [29]. In addition, the wires can store higher energy than the dots do and it is easier to control the growth quality of wires.

In terms of the above consideration, we are proposing a new super-gain nanostructure based on the InGaAs-GaAs-GaAsP material system. In the super-gain nanostructure, a special In_*x*_Ga_1−*x*_As quantum well with continuously-changed In-contents (*x*-values) that result in the corresponding band-gap to change in a continuous way is formed based on the strain-driven In-segregation effect [30, 31]. This ingeniously overcomes the difficulty in accurately controlling In-content of the In_*x*_Ga_1−*x*_As compound in the material growth. Meanwhile, the on-well InGaAs wires with different In-contents varying along the growth direction are also formed by means of the In-segregation effect and the growth orientation-dependent self-assembled multi-atomic step effect. The details of the super-gain nanostructure and its luminescence mechanism, optical properties as well as application in tunable laser diodes are characterized and investigated here for the first time. These are described in the following sections.

## 2 Results and discussion

### 2.1 Structural design

As an important link to the energy-band engineering of the super-gain nanostructure, the underlying interaction between hetero-dimensional quantum structures is paid more attention in the device design. In the material growth, the multi-atomic and periodic steps may be formed on the 200 nm-thick GaAs buffer layer based on the slightly-misoriented GaAs (001) substrate through the metal organic chemical vapor deposition (MOCVD) process [32–34]. This effect will be the foundation of constructing on-well InGaAs nanowires in the super-gain nanostructure. The InGaAs-GaAs-GaAsP material system is used as the core of the nanostructure here. It can generate higher emitting power at the waveband of 900 nm–1100 nm due to higher barriers and therefore is very useful in many areas [35–39]. For example, it can be used for generating terahertz (THz) waves [35], pumping the erbium-doped fiber amplifiers and fiber lasers [36–38], passively mode-locked fiber lasers as well as achieving high power of short-wavelength laser output (450 nm–550 nm) by combining second-harmonic generation (SHG) [39], etc. In order to obtain the InGaAs-based super-gain nanostructure, a 10 nm-thick InGaAs well with the initial composition of In_0.17_Ga_0.83_As is used in the nanostructure, where the higher 0.17 In-content is designed for the well to form a highly-strained crystal structure and ensure the strain-driven In-segregation effect to happen. It is important to forming the continuous variation of band-gap both in the well and in the wires. The InGaAs well is sandwiched by two 2 nm-thick GaAs strain-buffer-layers to reduce the strain difference between the well and the barrier due to a large lattice mismatch and to avoid serious deterioration of the crystal structure. Beyond the GaAs strain-buffer-layers are the 8 nm-thick GaAs_0.92_P_0.08_ barriers. The InGaAs-GaAs-GaAsP kernel is covered by the 2 μm-thick AlGaAs waveguide layers.

**Figure 1:**
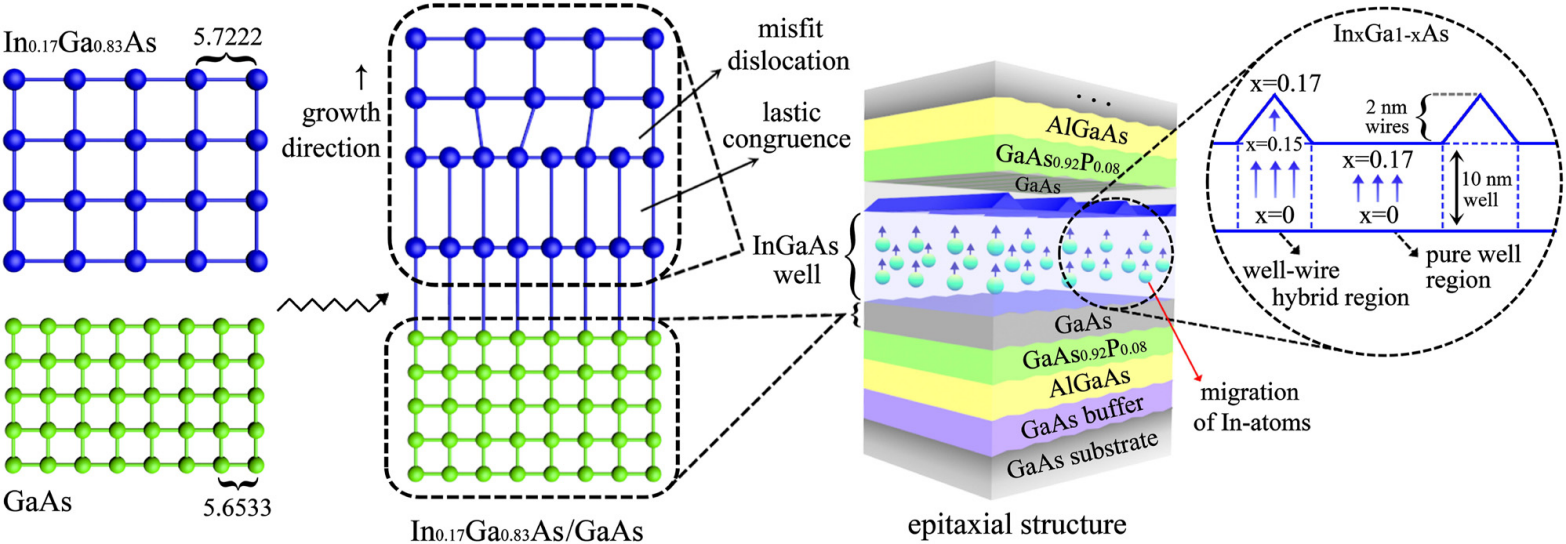
Schematic illustration of the super-gain nanostructure grown on the slightly-misoriented (001) GaAs substrate and its formation mechanism including the In-segregation and self-assembled multi-atomic step effects.

The formation mechanism of the super-gain nanostructure is illustrated in Figure 1. At the beginning of the In_0.17_Ga_0.83_As growth, the lattice constants of In_0.17_Ga_0.83_As and GaAs compounds are elastically coherent, that is, the larger In_0.17_Ga_0.83_As lattice is laterally compressed (perpendicular to the growth direction) to match the GaAs one and vertically stretched (along the growth direction) at the same time, as are illustrated by the left and the middle drawings in Figure 1. In terms of the energy balance theories, the strain will be absorbed coherently and no dislocation occurs in the InGaAs material, as long as the InGaAs strain-layer grown on the GaAs material is less than some thickness (the critical thickness) [40]. Otherwise, the dislocation will occur to relieve the strain due to the lattice mismatch in the InGaAs layer. It will become more apparent in a thicker InGaAs material grown on the GaAs buffer because of the strain accumulation. This process is accompanied by monotonic enlargement rather than abrupt alteration of the surface lattice [40–44]. It is generally convinced that In-atoms are easier to occupy the upper regions of the InGaAs layer than gallium (Ga) atoms due to their larger sizes in the migration process. In view of the kinetics, the migration length of the In-atoms is also much larger than that of the Ga-atoms. Thus, a part of In-atoms will move from the bottom to the top of the InGaAs layer so that the In-content increases continuously along the growth direction in the InGaAs layer, as is illustrated by the right drawing in Figure 1. It leads to the In-content reduced in the InGaAs compound and therefore the band-gap changed. This physical process is called the In-segregation effect, which occurs both in the well and in the wires of the super-gain nanostructure.

### 2.2 Modeling

For a complex optical system, theoretical simulation can provide effective routes for scientific research [45–47]. Here, we utilize the photonic integrated circuit simulator in 3D (PICS3D), a professional three-dimensional simulator designed for surface-emitting and edge-emitting laser diodes and other related waveguide photonic devices, to calculate the structural parameters, optical characteristics and optimize the model for designing the super-gain nanostructure. The epitaxial structure model is drawn in Figure 1, in which a certain amount of In-atoms will migrate along the growth direction due to the In-segregation effect and the multi-atomic step effect will lead to the on-well nanowires formed. Since the cross-section of the nanowires is approximately a triangle with a very small base-angle and a large top-angle, according to the atomic force microscopic (AFM) measurement result, it can be considered that there is a certain space between the effective nanowire areas. So the overall nanostructure model can be composed of two parts. One is the well-wire hybrid regions containing the well and the on-well wires. The other one is the pure well regions without the nanowires, as shown in the right drawing of Figure 1. In order to accurately construct the required nanostructure, we consider the In-content to vary from *x* = 0 to *x* = 0.15 in the In_*x*_Ga_1−*x*_As well with 10 nm in thickness and *x* = 0.15 to *x* = 0.17 in the In_*x*_Ga_1−*x*_As wires with 2 nm in height along the growth direction for the well-wire hybrid regions and the In-content to vary from *x* = 0 to *x* = 0.17 in the pure In_*x*_Ga_1−*x*_As well regions with 10 nm in thickness, as shown in Figure 1.

**Figure 2:**
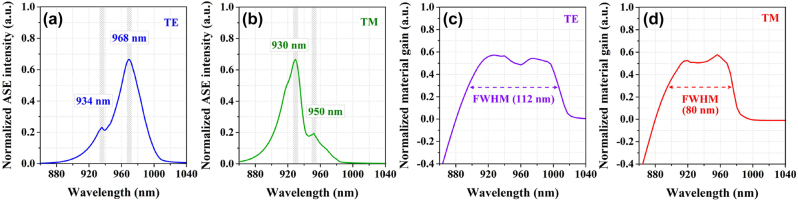
The calculated (a) TE and (b) TM ASE spectra based on the InGaAs super-gain nanostructure model. The calculated (c) TE and (d) TM gain spectra based on the same model.

With the above structural model, we calculate its amplified spontaneous emission (ASE) and optical gain spectra in both transverse electric (TE) and transverse magnetic (TM) polarization modes. The results are shown in Figure 2, where the injection peak-power is 15.9 W. The appearance of double peaks in all ASE spectra in Figure 2a and b is attributed to the combined emissions of the well-wire hybrid regions and the pure well regions. The TE ASE peaks are at 968 nm and 934 nm and the TM ASE peaks are at 950 nm and 930 nm. The interesting result is that both TE and TM gains in Figure 2c and d are remarkably broadened and flattened at top of the gain spectra due to this special design of the nanostructure. The full widths at half-maximum (FWHMs) of the gain spectra reach 112 nm and 80 nm, respectively, for the TE and TM modes at the injection peak-power of 15.9 W. These theoretical predictions on the super-gain features of the proposed nanostructure offer the possibility of achieving the real super-gain nanostructure for high performance of tunable laser diodes.

### 2.3 Preparation of experimental samples

The InGaAs super-gain nanostructure samples are fabricated according to the above design. The samples are grown on the GaAs (001) substrate in a slightly misoriented direction under a 100-mbar pressure and at the total flow rate of 13 slm using the aix200/4 MOCVD system. The growth rate of the materials is about 1 μm/h at a V/III ratio of 50. Meanwhile, a higher temperature of 650 °C is applied to increase the diffusion length of the In-atoms for redistributing the In-content in the InGaAs layer [48]. The composition of the epitaxial structure of the materials for the experimental samples is consistent with the theoretical design illustrated in Figure 1.

### 2.4 Structural characterization

**Figure 3:**
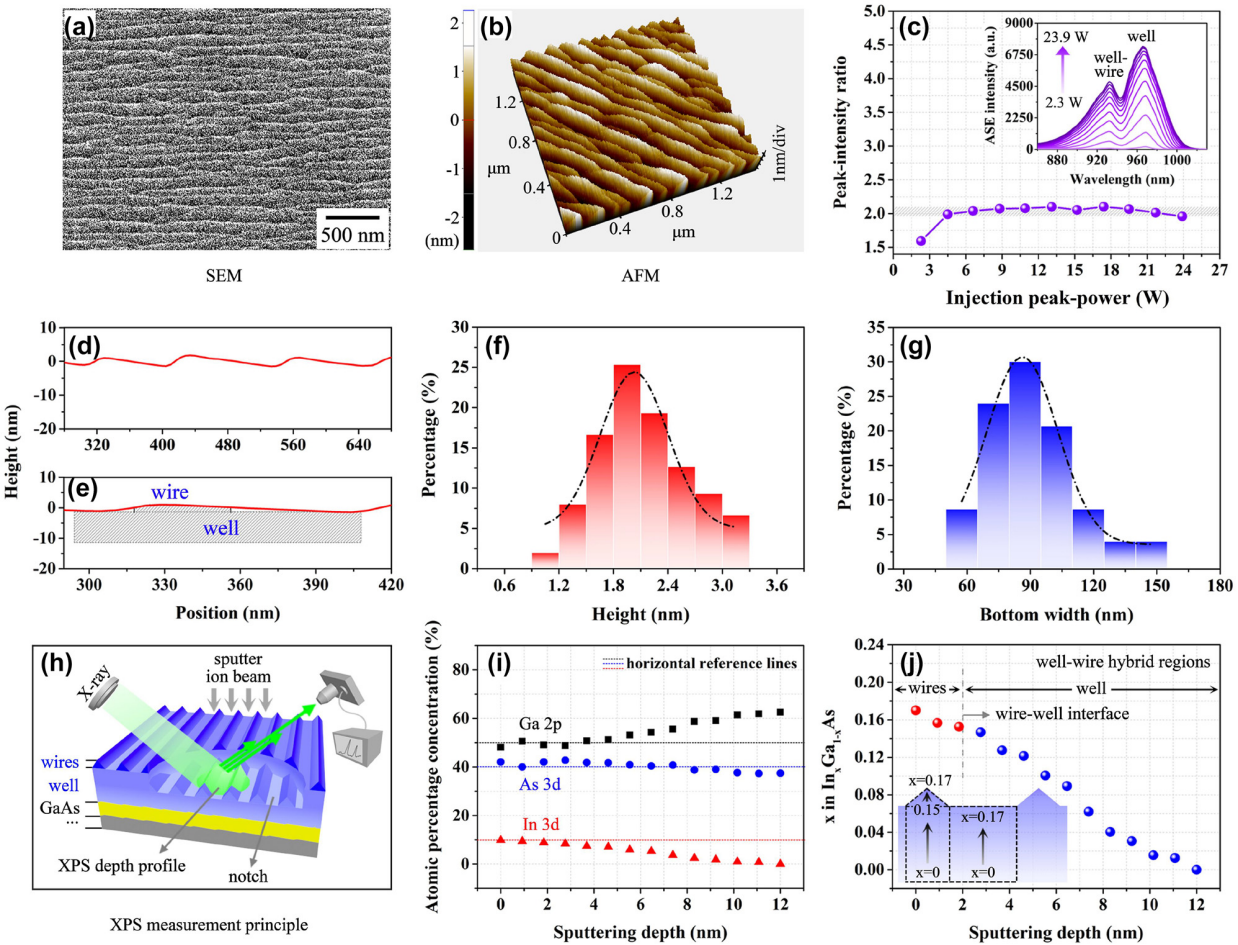
The morphologic measurement of InGaAs super-gain nanostructure surface: (a) top-view from the SEM measurement, (b) 3D-view from the AFM measurement. (c) The ratio of ASE peak intensities generated by the pure well regions and the well-wire hybrid regions as a function of injection power, where the inset is the unpolarized ASE spectra of the super-gain nanostructure from various injection power levels of 2.3 W–23.9 W. (d) The nanowire cross-section from line scanning perpendicularly to the wires and (e) the full width of a single nanowire on the well. (f) and (g) The statistical distributions of height (red pillars) and full width (blue pillars) of the wires in Gaussian fitting (chain lines) by measuring 150 wires at random. (h) Schematic diagram of the XPS measurement for atomic concentrations in the InGaAs super-gain nanostructure. (i) The measured atomic concentrations of Ga (blank squares), As (blue circles) and In (red triangles) elements at different ion-sputtering depths of the material. (j) The In-contents at different ion-sputtering depths of the material and the In-content distributions in the well-wire hybrid regions and the pure well regions of the nanostructure from the XPS measurement result.

The experimental super-gain nanostructure is examined and characterized using a special sample with the exposed InGaAs surface. It is fabricated under the same growth conditions by terminating its epitaxial growth process just after obtaining the complete InGaAs layer. Firstly, the nanostructure surface is morphologically observed and measured using an atomic force microscope (AFM, XE100) and a scanning electron microscope (SEM, Zeiss gemini300). The results are shown in Figure 3a–g. It is clearly confirmed by Figure 3a and b that the self-assembled nanowires are formed on the InGaAs well layer. The pure well regions and the well-wire hybrid regions in the super-gain nanostructure are approximately divided according to a ratio (*I*_well_/*I*_well-wire_) of two peak intensities of the unpolarized ASE spectrum (see the inset of Figure 3c), as the two peaks occur respectively from the above two types of regions and the peak intensities are proportional to luminescence areas. The peak-intensity ratio as a function of the injection power is calculated and shown in Figure 3c. The result in Figure 3c shows that the ratio, *I*_well_/*I*_well-wire_ converges on a stable value of 2 with increase of the injection power. Thus, *I*_well_/*I*_well-wire_ = 2 is taken as the criteria of dividing the pure well regions and the well-wire hybrid regions in the super-gain nanostructure. It is also confirmed by measuring the cross-section distribution of the on-well nanowires with line scanning perpendicularly to the nanowires, as is shown in Figure 3d. We notice from Figure 3d that the nanowire cross-section presents a triangle-like distribution with a very small base-angle and a large top-angle. It means that the effective areas of the on-well nanowires do not cover all well surface. Instead, they present an arrangement in a certain space, as shown in Figure 3e. The result approves of the model about the well-wire hybrid regions and the pure well regions, which is stated in the section of modeling. The measured nanowire sizes are statistically analyzed by taking 150 wires at random according to morphology. The results are shown in Figure 3f and g, where the wire height is between 1 nm and 3 nm and the full width of the wires is between 60 nm and 150 nm, which fit the Gaussian distribution centered at 2 nm and 87 nm well, respectively. Since the self-assembled nanowires on the well are not very regular in size and distribution, they will form slightly different energy-bands and light emission differences. As a result, the whole optical characteristics of the structure will be determined by the superposition or the statistical mean of all wire and well contributions. This should be also one of the reasons why the perfect super-gain spectrum can be formed from such a hybrid nanostructure.

Secondly, the In-content variation in the InGaAs layer is measured with the X-ray photoelectron spectroscopy (XPS, PHI Quantera II). This measurement can obtain the relative content of every element in a compound, according to the energy distribution of photoelectrons captured from the sample surface, when X-ray photons are irradiated. The measurement process is schematically illustrated in Figure 3h, in which a notch that is larger than the X-ray beam in diameter is cut by ion sputtering, and then all element atoms are detected by an X-ray beam at the notch bottom. The measurement process is completed step by step from top to bottom of the sample. In the measurement, the content of every kind of element atoms in the material is an averaged value related to the detection area. With the XPS measurement, the contents of different kinds of element atoms as a function of the material sputtering depth is obtained and shown in Figure 3i, where the energy levels of In 3d, Ga 2p and arsenic (As) 3d are selected for the XPS analysis. In Figure 3i, black square, blue circle and red triangle symbols indicate Ga, As and In concentrations in the nanostructure, respectively. Clearly, the In-content presents an approximately-linear reduction along the ion-sputtering direction, i.e. the linear increase along the material growth direction. On the contrary, Ga-content reduces along the material growth direction accordingly. There is little change for the As-content. These results are consistent with our theoretical analysis on the In-segregation effect. The type, content and distribution of elements are important indices for characterization of materials [37, 49, 50]. Since different characterization techniques have different pertinence in quantitative and qualitative analyses, element distribution evaluation, chemical state and molecular structure analyses, and so on, in the face of numerous material analysis technologies, it is necessary to select appropriate characterization tools, according to the material properties, material information to be obtained and the detection sensitivity to be met, etc. In our research, XPS is a suitable measurement technique to obtain the material information we need.

The In-content distribution in the InGaAs well-wire hybrid regions can be divided into two parts along the growth direction, according to the wire height and the well width, as shown in Figure 3j, in which the small balls are the XPS measurement data obtained at different ion-sputtering depths of the InGaAs material. The result in Figure 3j shows that the In-contents are monotonically changed approximately from 0 to 0.15 in the well and from 0.15 to 0.17 in the wires along the material growth direction for the well-wire hybrid regions according to the data in Figure 3i. Thus, it can be estimated that the In-content varies approximately from 0 to 0.17 in the pure well regions along the growth direction. Since the In-content alteration will cause the InGaAs band-gap to change for the well and the wires, in terms of the energy band theory [51], a kind of self-assembled InGaAs well-wire complex nanostructure with the continuously-changed band-gap is formed.

### 2.5 Spontaneous emission characteristics

**Figure 4:**
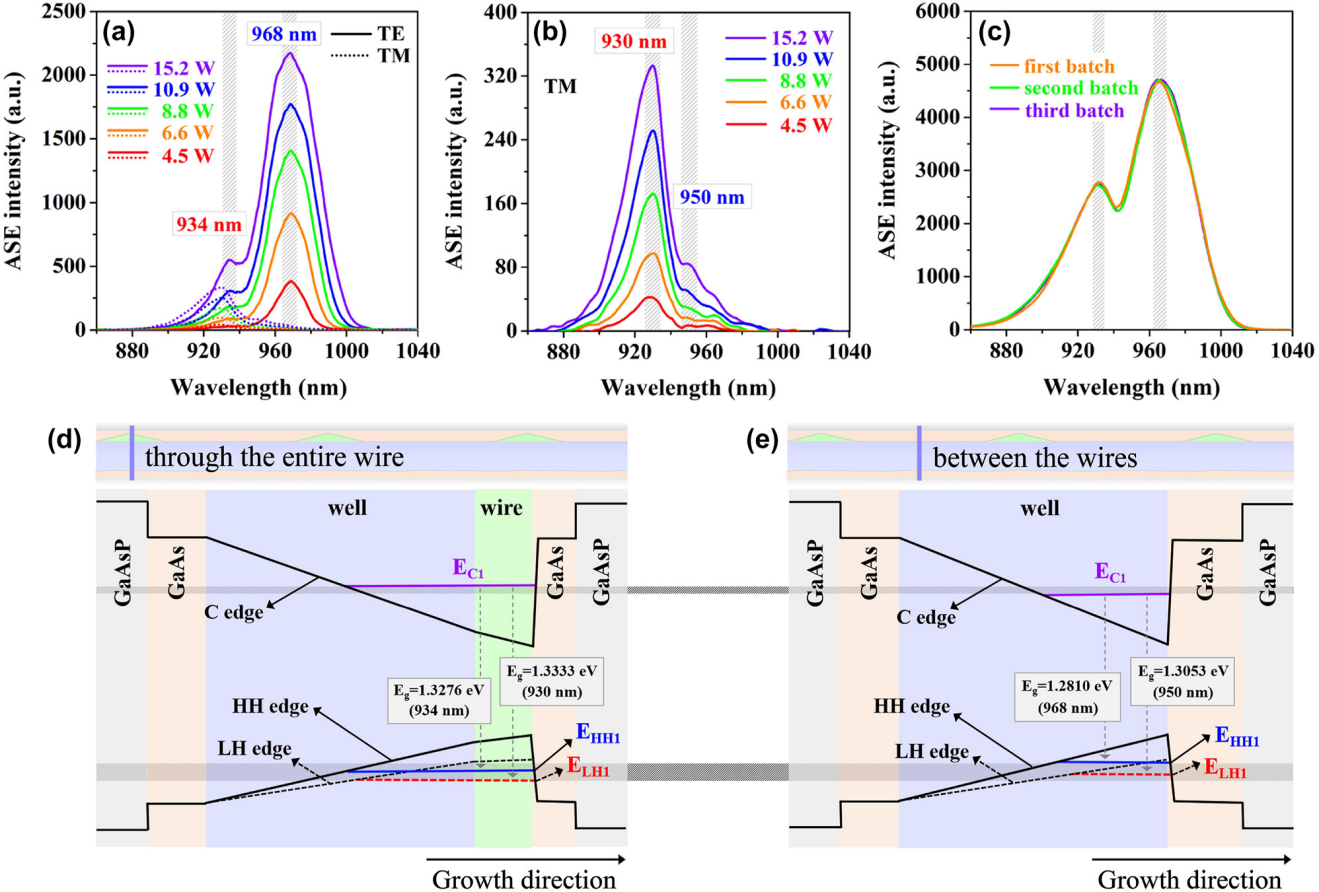
Spontaneous emission characteristics and energy-band structures of super-gain nanostructure. (a) The measured ASE spectra of the TE and TM polarization modes of the InGaAs super-gain nanostructure with various injection peak-power levels of 4.5 W–15.2 W at room temperature. (b) The magnified view of the TM-polarized ASE spectra in (a). (c) The unpolarized ASE spectra of three different batches of super-gain nanostructure samples fabricated under the same growth conditions and the injection peak-power is 12.5 W. (d) and (e) Energy-band diagrams of the self-assembled InGaAs well-wire hybrid regions and the pure well regions without the on-well nanowires, respectively.

The polarized ASE intensities of the super-gain nanostructure are detected from the two facets of an edge-emitting sample at room temperature, where a beam of 808 nm pulsed-laser (LWIRL808-40W-F) with a pulse width of 20 ms and a frequency of 1 Hz is used to optically pump the sample from the top in the experiment. The measurement results are shown in Figure 4a and b. It is clearly seen from Figure 4a and b that the double peaks appear in all ASE spectra, which are located at 968 nm and 934 nm in the TE-polarized ASE spectra, and at 950 nm and 930 nm in the TM-polarized ASE spectra. These peaks rise monotonically with increasing injection peak-power levels from 4.5 W to 15.2 W. The stronger TE emissions than the TM ones are attributed to the dominant compressive-strain in the nanostructure. Thus, we first focus on the TE emission feature analysis. The major peak at 968 nm is apparently higher than the second one at 934 nm in all the TE-polarized ASE spectra. This is because the major peak is generated from the pure well regions and the secondary one occurs from the well-wire hybrid regions. In addition, for the well-wire hybrid regions, the peak is higher at 934 nm in the TE-polarized ASE spectra than at 930 nm in the TM-polarized ASE spectra, as the compressive strain exists in these regions. And the peak is much higher at 968 nm in the TE-polarized ASE spectra than at 950 nm in the TM-polarized ASE spectra for the pure well regions, as shown in Figure 4a. As there are still obvious bi-peaks and the peak intensity at 930 nm is much higher than that at 950 nm in the TM-polarized ASE spectra, even if the injection power is at a very low level, we can conclude that the secondary peaks at 934 nm in the TE-polarized ASE spectra and at 930 nm in the TM-polarized ASE spectra do not come from the emissions of the second energy level of the pure well regions.

In addition, the unpolarized ASE spectra of three different batches of super-gain nanostructure samples fabricated under the same growth conditions are measured to examine the repeatability of the super-gain nanostructure performance. The result is shown in Figure 4c, where the same injection peak-power of 12.5 W is applied. It is clearly seen from Figure 4c that the major features of the three spectra including peak wavelengths, bandwidths and spectral configurations match each other very well. This proves that the described super-gain characteristics can be repeatedly obtained well with the described nanostructure of the material for the actual application.

The luminescence mechanism of the super-gain nanostructure is revealed by the energy-band analysis. The nanostructure can be composed of two main structural parts: the self-assembled well-wire hybrid regions and the pure well regions without the on-well nanowires. The energy band structures of these two parts are drawn in Figure 4d and e, respectively. Since the band-gap is continuously changed within both well and wires due to the In-segregation effect, the band-edges will be inclined. The GaAs buffers and the GaAs_0.92_P_0.08_ barriers lead to steps of the barriers in the energy-band structure. The slight tilt of the right barrier in the band structure is because a small minority of In-atoms may enter into the GaAs buffer that overlies the InGaAs surface due to the In-segregation effect so that there is an extremely-thin transition layer between the InGaAs layer and the GaAs buffer. According to the relation of lattice mismatch and strain of the semiconductor materials [52], we can conclude that both of the InGaAs well-wire hybrid regions and the pure InGaAs well regions are in the compressive strain status for the super-gain nanostructure. In terms of the solid theory, the compressive strain in the material makes the heavy-hole (HH) band to be higher than the light-hole (LH) band in the valence band. Thus, the carrier recombination between the first conduction band (C1) and the first heavy-hole valence band (HH1) is dominant [53–55], which produces the TE-polarized emission. In addition, the ASE peak from the well-wire hybrid regions will have a blue-shift relative to the ASE peak of the pure well regions. This is because a broader band-gap is formed in the well-wire hybrid regions than in the pure well regions due to the further dimensional confinement of the nanowires and the lower In-content in the well, as illustrated in Figure 4d and e.

The particularity of the quantum in the super-gain nanostructure lies in that the direct coupling of a two-dimensional quantum well and one-dimensional nanowires leads to a sort of hybrid quantum structure, in which the carriers are more strongly confined than in the pure well due to the wire role. Meanwhile, the density-of-states of electrons is also more concentrated in this hybrid quantum structure due to the combined wires. The increase of quantum confinement in the new structure will strongly modify the position of energy levels, leading to the redistribution of electrons in the conduction band. This is because the C1 energy level in the wires occupies a higher energy position than in the well so that a unified C1 energy level that is higher than C1 level in a pure well and lower than that in the wires will be formed in the well-wire hybrid regions along with a unified Fermi-level from the combined role of both wires and well. Similarly, the unified HH1 energy level and LH1 energy level are also formed, as shown in Figure 4d. These quantum effects largely change the traditional gain spectrum to a super-gain one.

**Figure 5:**
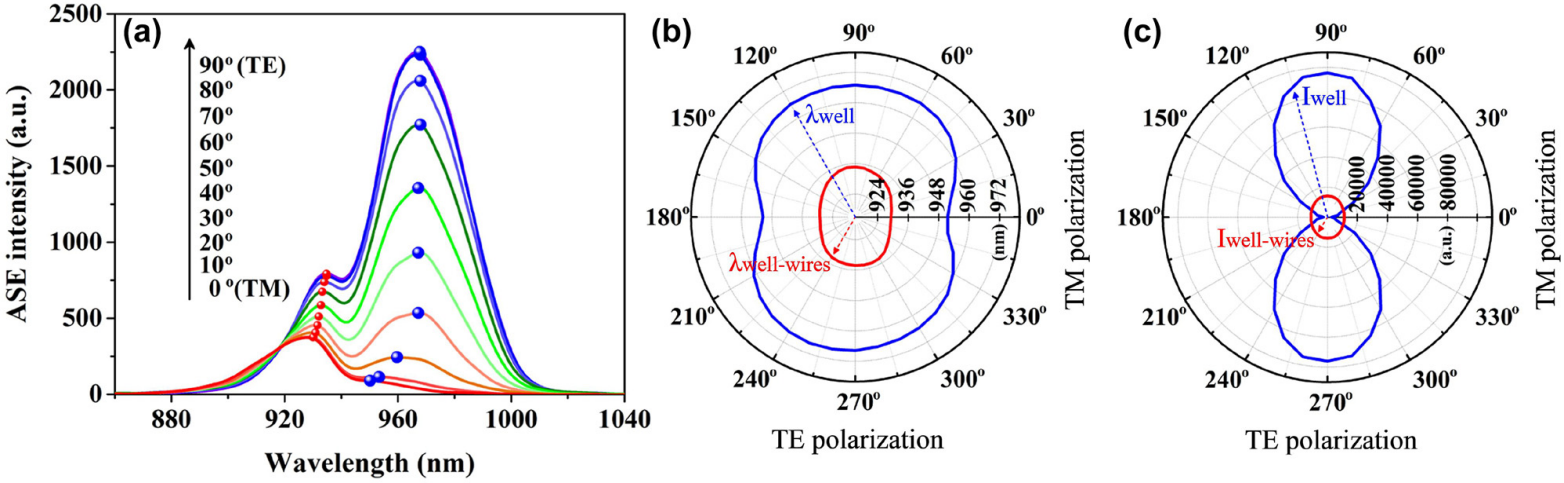
Polarization feature of super-gain nanostructure. (a) The shift of ASE peaks due to varying the polarization from TM to TE modes in the InGaAs super-gain nanostructure. (b) The peak wavelengths and (c) the peak intensities of ASE spectra as the functions of polarizer angles for the well-wire hybrid regions and the pure well regions.

The polarization anisotropy of the spontaneous emissions of the super-gain nanostructure is investigated by measuring the TE- and TM-polarized ASE intensities of an edge-emitting sample at different polarizer angles, which are determined by rotating the polarizer under a fixed injection peak-power of 15.2 W. The results are shown in Figure 5, in which we define the TM polarization as the 0° (180°) polarizer angle, i.e. it takes the direction normal to the sample surface and the TE polarization as the 90° (270°) polarizer angle, i.e. it is corresponding to the in-plane direction. The result in Figure 5a shows that both of ASE peaks rise when switching the polarizations from TM to TE modes, i.e. rotating the polarizer from 0° to 90°, and meanwhile, the main peak moves from 950 nm to 968 nm and the secondary peak moves from 930 nm to 934 nm. The red-shift of the ASE peaks due to switching the polarization from TM to TE modes in Figure 5a is because of the compressive strain role in the well-wire hybrid regions and the pure well ones.

In order to illustrate more clearly the polarization feature of the super-gain nanostructure, the wavelengths and intensities of the ASE peaks as the functions of polarizer angles are drawn in Figure 5b and c, respectively, in which blue and red loops represent wavelength and intensity variations of the ASE peaks due to switching between TM and TE modes in the pure well regions and the well-wire hybrid regions, respectively. The maximum and the minimum points of the blue loops are corresponding to the main peaks at 968 nm and 950 nm, respectively, while the maximum and the minimum points of the red loops are corresponding to the second peaks at 934 nm and 930 nm, respectively. The polarization degree (*ρ*), which is defined as *ρ* = (*I*_TE_ − *I*_TM_)/(*I*_TE_ + *I*_TM_) [56, 57], is 0.95 for the emissions from the pure well regions and 0.12 for the emissions from the well-wire hybrid regions. The smaller polarization degree of 0.12 indicates the better strain release inside the well-wire hybrid regions than in the pure well regions.

### 2.6 Gain characteristics

**Figure 6:**
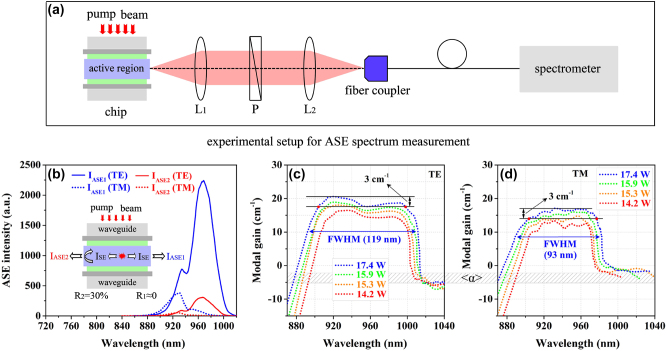
Gain characteristics of super-gain nanostructure. (a) Experimental setup for ASE spectrum measurement of the edge-emitting sample. (b) Principle of the gain measurement with the ASE spectra detected from both facets of an optically-pumped edge-emitting super-gain sample. (c) TE modal gains and (d) TM modal gains with various injection peak-power of 14.2 W–17.4 W.

The optical gain is a key factor determining laser diode performance. It can be obtained by measuring ASE spectra from both facets of an edge-emitting device and using the following equation [58]. Considering that the optimized experimental device and optical system are important factors of obtaining stable signal output [59–61], the experimental setup for ASE spectrum measurement of the super-gain nanostructure is designed and described in Figure 6a, in which two lenses of *L*_1_ and *L*_2_ and a linear polarizer P are used for collection of the emissions of different polarization modes. The optical signals are detected using a spectrometer (HR4000CG-UV-NIR) coupled with a fiber. In addition, the gain measurement principle is briefly illustrated in Figure 6b. The experimental sample is processed to be an in-plane configuration with 1.5 × 1 mm^2^ in size. The right-facet of the sample is coated with the transmittance of *T* = 99.99% and the left-facet is uncoated. Hence, the right-facet reflectivity is *R*_1_ ≈ 0 and the left-facet reflectivity is *R*_2_ = 30%, which is determined by material indices.

(1) $$G=\frac{1}{L}\ln\frac{\left(1-R_{2}\right)I_{\text{AES}1}-I_{\text{AES}2}}{R_{2}\times I_{\text{AES}2}}$$

where *L* = 1.5 mm is the single-pass length of light propagating through the sample. *I*_ASE1_ denotes the ASE intensity detected from the *R*_1_-facet, which is the intensity superposition of the single-pass light through *R*_1_-facet and the round-trip light reflected by *R*_2_-facet from the same point source. *I*_ASE2_ denotes the ASE intensity detected from the *R*_2_-facet, which is a part of the single-pass light due to the *R*_2_-facet reflection. The gain measurement results are shown in Figure 6c and d. The different injection peak-power levels of 14.2 W, 15.3 W, 15.9 W, and 17.4 W are applied.

**Figure 7:**
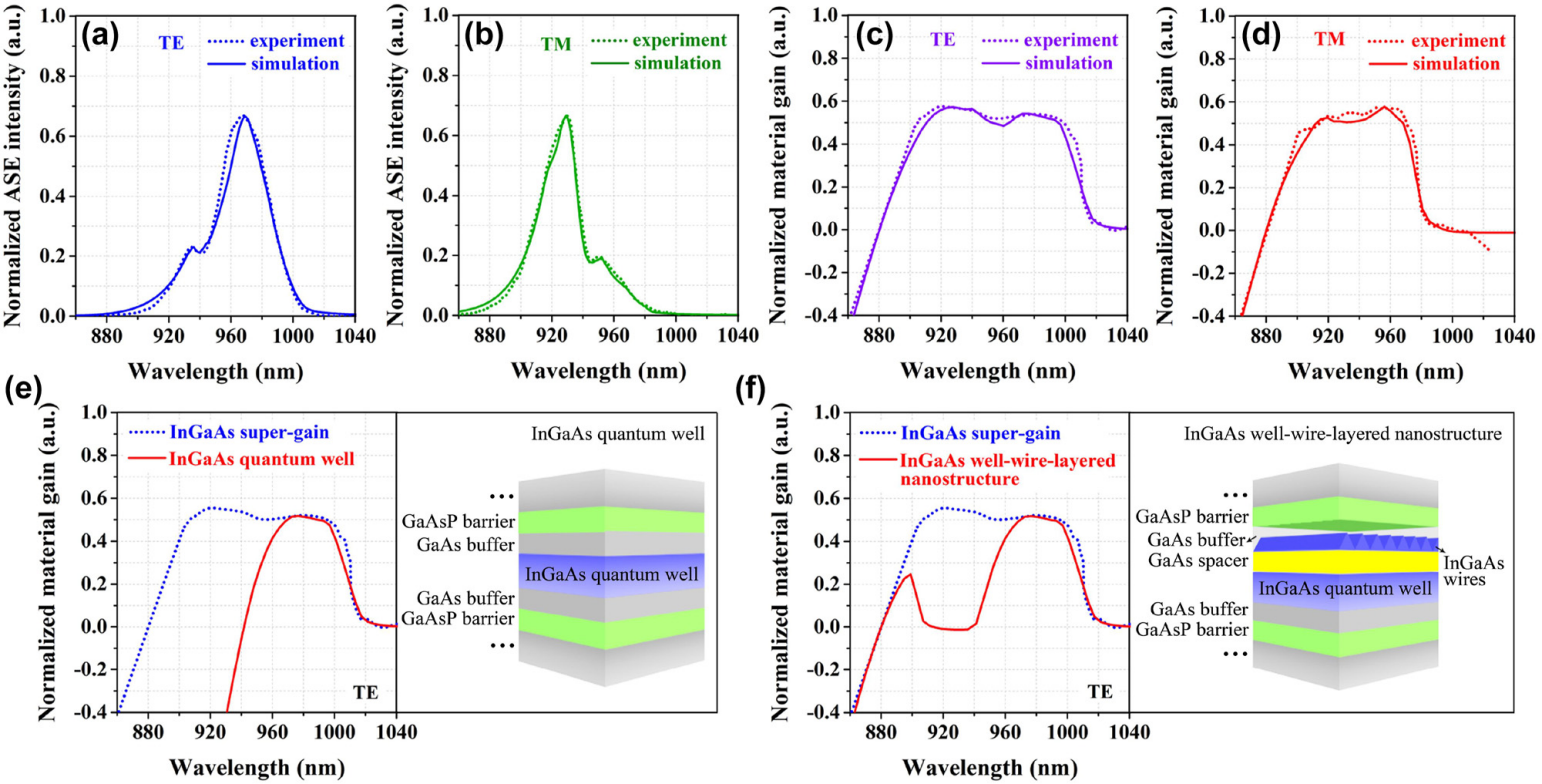
The comparison of (a) TE and (b) TM ASE spectra of the InGaAs super-gain nanostructure from experiment and theoretical model. The contrast of (c) TE and (d) TM gain spectra of the InGaAs super-gain nanostructure from experiment and theoretical model with the injection peak-power of 15.9 W. (e) The structural diagram of the typical InGaAs well and its TE gain spectrum (red curve). (f) The structural diagram of the InGaAs well-wire-layered nanostructure and its TE gain spectrum (red curve). The blue dot curves in (e) and (f) are the same gain spectrum of the InGaAs super-gain nanostructure.

**Figure 8:**
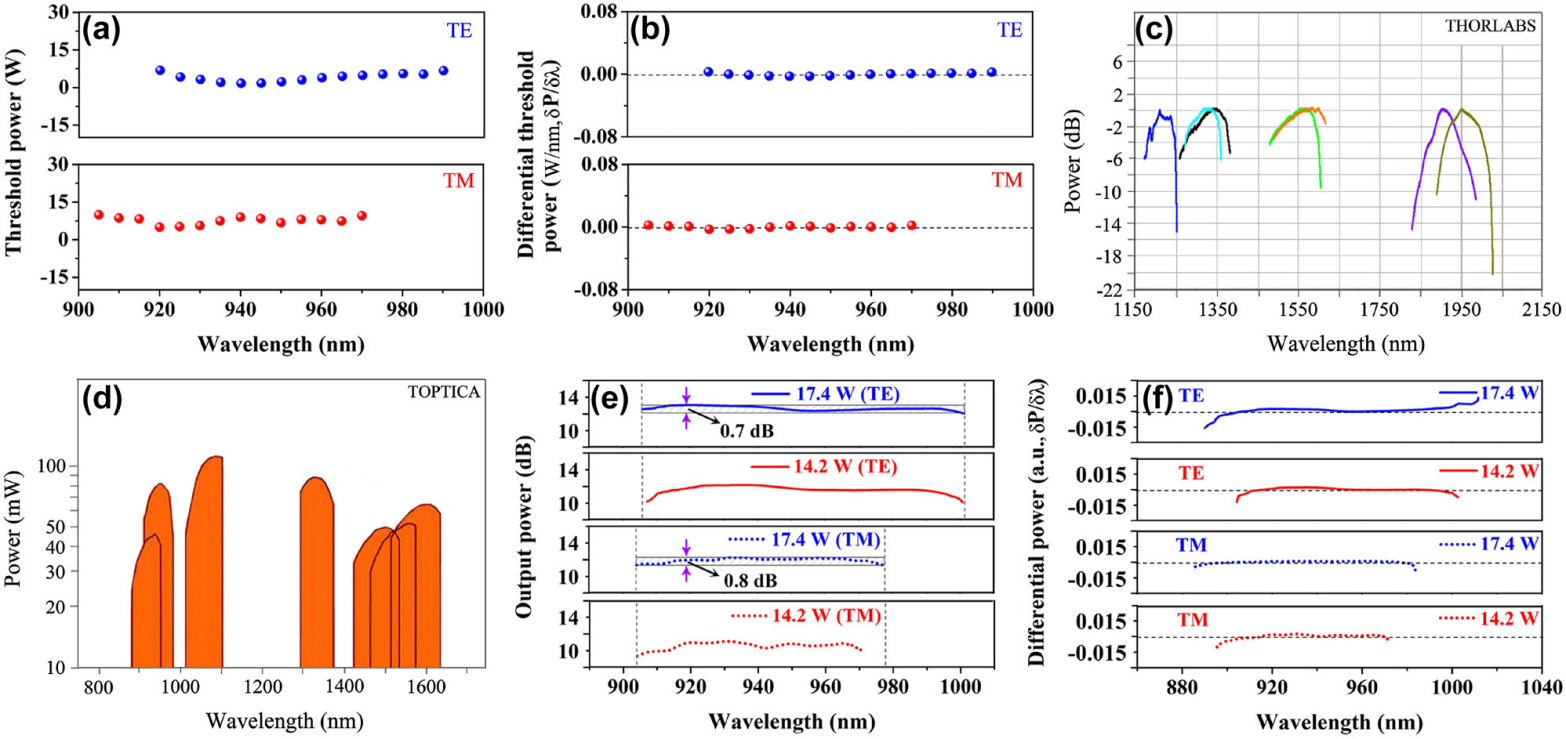
Wavelength-related threshold and output power characteristics of super-gain nanostructure. (a) Threshold injection power as a function of wavelength for TE and TM polarization modes with the InGaAs super-gain nanostructure as the laser medium. (b) The differential of threshold injection power as a function of wavelength for different polarization modes. Spectral power as a function of wavelength of (c) tunable laser diode products (Thorlabs, model: TLK-L) and (d) tunable semiconductor laser products (Toptica, model: CTL), respectively. (e) Polarized spectral output power as a function of wavelength with various injection power levels. (f) The differential of polarized output power as a function of wavelength with different injection power levels.

The results of gain measurement reveal the following important information. First of all, the averaged internal loss is <*α*> = ∼3 cm^−1^. Secondly, the TE-polarized gain spectrum reaches both 119 nm in FWHM and a very small fluctuation of <3 cm^−1^ over a wavelength range of 94 nm of the top under the injection peak-power of 17.4 W. Meanwhile, the TM-polarized gain spectrum also reaches 93 nm in FWHM and a small fluctuation of <3 cm^−1^ over a wavelength range of 73 nm of the top under the same injection power. In addition, both TE and TM modes obtain comparable and nearly uniform gain spectra in a wide wavelength range of 73 nm (from 904 nm to 977 nm). This enables both TE and TM lasing in this wide wavelength range for a strain laser device. These gain data reflect the super-gain features to be achieved for high performance of tunable laser diodes. The experimental results are perfectly consistent with our theoretical prediction from modeling of the super-gain nanostructure, as is shown Figure 7a–d, in which the experimental ASE and gain spectra match the theoretical results very well under the same conditions. Therefore, it can be concluded that an InGaAs super-gain nanostructure consisting of the self-assembled well-wire hybrid regions and the pure well regions with the continuously-changed band-gap is successfully realized from our experiment.

In order to further ensure the superiority of the InGaAs super-gain nanostructure, we also calculate the gain characteristics of two typical types of nanostructure models by means of PICS3D, as a comparison. One is the InGaAs-GaAs-GaAs_0.92_P_0.08_ quantum well with the In-segregation effect, as is shown in Figure 7e, in which a 10 nm-thick InGaAs well with the In-content varying from *x* = 0 to *x* = 0.17 along the growth direction is used. In this case, the TE-polarized gain spectrum under the same injection conditions is calculated. The result is plotted in Figure 7e (red curve), where the blue dot curve is the TE gain spectrum of the super-gain nanostructure. The other one is the InGaAs well-wire-layered nanostructure, as shown in Figure 7f, in which a 10 nm-thick well layer and a 2 nm-thick wire layer are separated by a GaAs spacer. The In-content varies from *x* = 0 to *x* = 0.17 in the In_x_Ga_1-x_As well along the growth direction due to the In-segregation effect and there is a fixed In-content of *x* = 0.17 within the InGaAs wires. The TE-polarized gain spectrum of this structure is calculated under the same injection conditions. The result is plotted in Figure 7f (red curve), where the blue dot curve is the TE gain spectrum of the super-gain nanostructure. The results in Figure 7e and f indicate clearly that it is difficult for the two typical nanostructures to achieve the ideal super-gain features.

### 2.7 Wavelength-related threshold and output power characteristics

The threshold injection power or carrier density is an important factor of measuring the laser diode performance. In order to look at the wavelength-related threshold injection power behavior of the super-gain tunable laser diode, the threshold gain <*G*>_th_ is calculated by the following equations [62]

(2) $${\left\langle G\right\rangle }_{\text{th}}=\left\langle \alpha \right\rangle +\alpha_{\text{m}}$$

and

(3) $$\alpha_{\text{m}}=\frac{1}{2L}\ln\left(\frac{1}{r_{1}r_{2}}\right)$$

where <*α*> is the averaged internal loss factor, which is <*α*> = ∼3 cm^−1^ from Figure 6c and d. Substituting *r*_1_ = 0.99, *r*_2_ = 0.9 and *L* = 1.5 mm into the above equations yields the threshold gain of <*G*>_th_ = ∼3.38 cm^−1^.

Therefore, the threshold injection power, *P*_th_ (*λ*) can be obtained by increasing the injection power to make the gain, *G* (*λ*) to match <*G*>_th_ for each wavelength in the gain bandwidth. The threshold injection power as a function of wavelength for different polarization modes are plotted in Figure 8a. The result shows that the threshold injection power fluctuates only a little in a wide spectral range of 70 nm for the TE mode and of 65 nm for the TM mode. The differentials of the threshold power in both TE and TM modes are plotted in Figure 8b, in which the differential values are about zero. This result indicates that the threshold injection power is almost independent of the wavelength change from 920 nm to 990 nm for the TE mode and from 905 nm to 970 nm for the TM mode if the super-gain nanostructure is used as the laser medium. This will greatly improve the tunable laser diode performance at the edge wavelengths. While it is hardly possible to achieve these using the traditional quantum-confined nanostructures as the laser medium.

The tunable wavelength range and uniformity of the spectral output power are two other important indices of measuring the tunable laser diode performance. Throughout the existing tunable laser diode products, not only is the tunable wavelength range very limited for a single laser device, but also the products show the poor uniformity of spectral output power under a fixed injection power level or carrier density. The specifications of some tunable laser diode products are presented in Figure 8c and d, in which each curve reflects a single laser diode performance. It is clearly seen from these data that the spectral output power falls severely at both ends of the tunable bandwidth.

In terms of the laser theory, the material gain (*g*) and threshold current density (*J*_th_) can be expressed by the following formulas, respectively [63].

(4) $$g=\frac{A\eta_{i}\tau_{sp}}{ed}\left(J-J_{tr}\right)$$

(5) $$J_{th}=J_{tr}+\frac{ed}{\Gamma \eta_{i}\tau_{sp}A}\left[\left\langle \alpha \right\rangle +\frac{1}{2L}\ln\left(\frac{1}{r_{1}r_{2}}\right)\right]$$

Here, *A* is a constant, *η*_*i*_ represents the internal quantum efficiency, and *τ*_sp_ is radiative recombination lifetime. In addition, *e* denotes the elementary charge and d is defined as the thickness of the active layer. *J* and *J*_tr_ represent the current density and transparency current density, respectively. Г is defined as the confinement factor. Furthermore, <*α*> and *L* (1.5 mm) denote the average internal loss of materials and the cavity length, respectively. *r*_1_ and *r*_2_ represent the reflectivity of the cavity mirrors, which are taken as *r*_1_ = 0.99 and *r*_2_ = 0.9 in the calculation. Therefore, the current density above the threshold can be written as:

(6) $$J-J_{th}\propto \left[\Gamma g-\left\langle \alpha \right\rangle -\frac{1}{2L}\ln\left(\frac{1}{r_{1}r_{2}}\right)\right]$$

In addition, the modal gain [*G*(*λ*)] of the laser medium and the spectral output power [*P*_out_(*λ*)] can be expressed as:

(7) $$G\left(\lambda \right)=\Gamma g-\left\langle \alpha \right\rangle$$

(8) $$P_{\text{out}}\left(\lambda \right)\propto J-J_{th}$$

So the following relationship exists:

(9) $$P_{\text{out}}\left(\lambda \right)\propto \left[G\left(\lambda \right)-\frac{1}{2L}\ln\left(\frac{1}{r_{1}r_{2}}\right)\right]$$

By substituting the gain data in Figure 6c and d into the Eq. (9), the output power with the InGaAs super-gain nanostructure for the laser diode can be worked out, as is shown Figure 8e and f. The result is amazingly perfect, as the almost identical output power over a very wide wavelength-tuning range can be achieved under the fixed injection power. The data in Figure 8e show that the max power fluctuation in the TE polarization mode is <0.7 dB throughout the wavelength range of 95 nm (from 906 nm to 1001 nm), where the fixed injection peak-power of 17.4 W is applied, while the max power fluctuation in the TM polarization mode is <0.8 dB throughout the wavelength range of 74 nm (from 904 nm to 978 nm) under the same injection power. The corresponding differential power, *δP*/*δλ* is calculated and plotted in Figure 8f. The result indicates once more that the spectral output power in both of TE and TM polarization modes is very uniform due to the very low differential power over the whole tunable wavelength range. This is a very significant to the development of high performance of tunable laser diodes. It is attributed to the realization of the super-gain nanostructure.

With the great promise in broadening the field of photonic applications and effectively promoting the development of semiconductor optoelectronic devices, the nanomaterials have attracted much attention currently [45, 50]. Based on the above work, the super-gain nanostructure with the ultra-wide and uniform TE and TM gain spectra not only brings a great chance for developing high performance of tunable laser diodes, but also provides many new inspirations for improving other performances of optoelectronic devices. For example, it is popularly known that thermal detuning between the gain peak and the cavity mode always happens and thus leads to a considerable carrier loss for single-frequency light-emitting diodes working at high temperature. This problem has not been solved well so far, which is related to the fact that the nanostructures of materials with a traditional single-peak gain spectrum are usually used as gain media. However, the above analysis shows that the temperature-insensitive gain-cavity alignment over a wide temperature range can be achieved with the super-gain nanostructure, as the gain fluctuation within an ultra-wide effective-bandwidth is very small. Therefore, it will be of great significance in overcoming the thermal gain-shift effect in quantum-confined single-frequency laser diodes. This significantly improves the device efficiency at high temperatures. Moreover, the well-wire interface effect is another interesting point to study. It enables a smaller polarization degree to be obtained in the well-wire hybrid regions of the super-gain nanostructure. This is observed in our experiment. It allows the achievement of depolarization in the strained devices, and therefore, brings a brand-new chance for developing polarization-insensitive and broadband optoelectronic devices. These potential advantages make the super-gain nanostructure to show great application prospects. The relevant investigations are in progress in our laboratory, as well.

## 3 Conclusions

In summary, we have reported a new InGaAs super-gain nanostructure in this paper, which is constructed based on the self-assembled well-wire complex energy-band engineering. With the In-segregation and the growth orientation-dependent on-GaAs multi-atomic step effects, the special well-wire hybrid regions and the pure well regions with continuously-changed band-gap are formed in the nanostructure. Through the control of material composition, content, structural dimension, size, thickness and growth conditions, as well as the utilization of self-assembling growth mechanism and direct coupling of one-dimensional wires and two-dimensional well, the complex energy-band structure of this super-gain nanostructure is effectively adjusted and the gain extends almost equally in the short-wave direction, compared with the traditional quantum well. Our theoretical modeling and experimental investigation on luminescence mechanism and optical characteristics of the super-gain nanostructure show that such a super-gain nanostructure can achieve the distinguished ultra-wide and top-flat super-gain spectra. It therefore demonstrates the great value in overcoming the shortcoming of the existing tunable semiconductor laser products and developing high performance of tunable laser diodes with the ultra-wide tunable wavelength range and the uniform spectral output power. In addition, the hybrid of hetero-dimensional quantum structures will also bring some particular physical properties, which provides an effective scheme to improve and control the physical properties and broaden the application fields of low-dimensional quantum nanostructures of optoelectronic devices.
